# Bilateral neglected facial invasive squamous cell carcinoma in an 89-year-old man, complete excision and primary reconstruction by cervicofacial flap, case report and review of current reconstruction approaches

**DOI:** 10.1016/j.ijscr.2025.111873

**Published:** 2025-08-30

**Authors:** Amin Rezazadeh, Ali Samady Khanghah, Sonia Sharifi Namin

**Affiliations:** a Department of Plastic and Reconstructive Surgery, School of Medicine, Ardabil University of Medical Sciences, Ardabil, Iran; b Department of Surgery, School of Medicine, Ardabil University of Medical Sciences, Ardabil, Iran; c Department of Pathology, School of Medicine, Ardabil University of Medical Sciences, Ardabil, Iran

**Keywords:** Squamous cell carcinoma, Parotid, Cervicofacial flap

## Abstract

**Introduction and importance:**

Non-melanoma skin neoplasms generally account for more than 80 % of occur in people in their 7th decade of life or higher, and increasing prevalence has made them a global concern. Squamous cell carcinoma (SCC) is the first step of the group of neoplasms mentioned above. An appearance of typical cutaneous squamous cell carcinoma (CSCC) is a non-healing, usually progressively enlarging erythematous papule, plaque, or ulcer. For cosmetic reasons, the surgeon should excise such lesions and then schedule second or third operations for flap-mediated reconstructions.

**Case presentation:**

After reporting a novel case of bilateral neglected facial invasive SCC affecting the parotid region in an 89-year-old man whose treatment with a semi-therapeutic palliative approach, we have conducted a review of facial CSCC from the aspects of epidemiology, staging, and therapeutic management.

**Clinical discussion:**

Despite mucosal SCC, cutaneous ones rarely metastasized, varying from 0.5 to 10 %. Subdividing primary cutaneous SCC into low and high-risk types has been of interest over the last decade since they behave differently regarding regional metastasis, resulting in prognosis differences. Most cutaneous SCC includes previously untreated small (less than 2 cm), thin (less than 2 mm), and well-differentiated cases.

**Conclusions:**

The surgeons should weigh against the outcome of their approach. For instance, in the case of older people and the risk of multiple general anesthesia, a palliative approach can be considered.

## Highlights

• Facial skin tumors should be approached with the measurements of cutaneous cancers rather than head and neck neoplasms.
• Non-melanoma skin cancers account for more than 80% of occur in people aged 60 years or higher.
• Primary cutaneous squamous cell carcinomas are subdivided into low and high-risk types.
• Most cutaneous SCC includes previously untreated small (less than 2 cm), thin (less than 2 mm), and well-differentiated cases, and nodal metastasis is rare.
• Local excision in the large group of low risk can be sufficient and curable.

## 1 Background

Facial SCC is part of the head and neck squamous cell carcinoma (HNSCC) group, which includes malignancies of the oral cavity, pharynx, hypopharynx, larynx, nasal cavity, and salivary glands.

It is better to approach the facial skin area with the measurements of cutaneous cancers. Non-melanoma skin cancers account for more than 80 % of occur in people aged 60 years or higher [1]. Besides aesthetic issues, the nonignorable role of organs located in the head and neck region in facilitating respiration and swallowing, as well as filtering and humidifying the air, makes HNSCC a significant health concern worldwide. The increasing incidence of HNSCC in many countries, especially among younger populations with a predicted 30 % annual increase in incidence by 2030, raises a global concern [2]. Based on an Australian series study, CSCCs commonly develop in direct sun exposure areas, with a tendency to the head and neck regions. According to this study, analyzing the anatomical distribution of 1219 head and neck CSCCs revealed the most common sites were the nose (20.0 %), cheek and maxilla (19.8 %), and ear (18.2 %) [3]. An appearance of a typical CSCC is a non-healing, usually progressively enlarging erythematous papule, plaque, or ulcer [4]. Here, we are reporting a case of a rapidly growing bilateral neglected facial invasive CSCC in an 89-year-old farmer man who had reached over 4 cm in diameter over seven months. This work has been reported in line with the SCARE criteria [5].

## 2 Case presentation

**Fig. 1:**
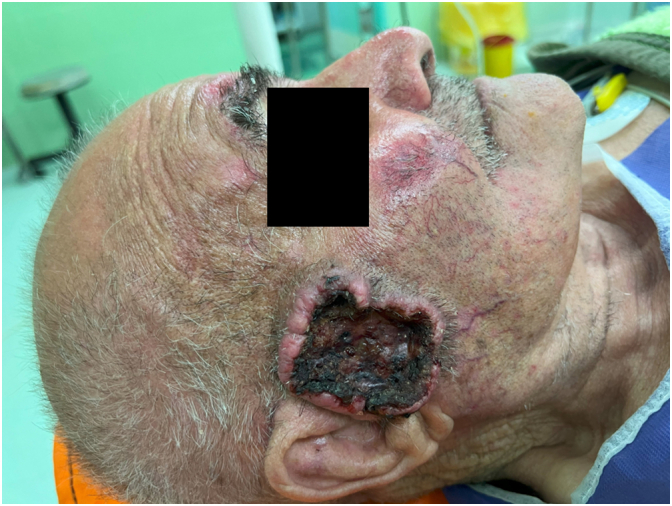
The right-side lesion involving also the auricle.

**Fig. 2:**
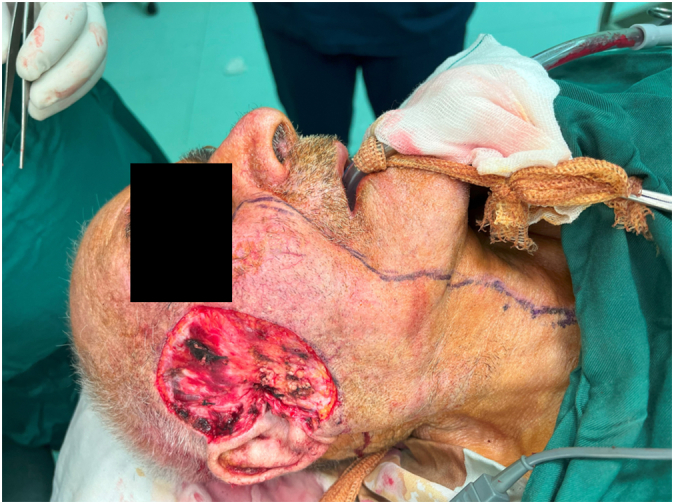
Excision of the right-sided lesion with superficial parotidectomy.

**Fig. 3:**
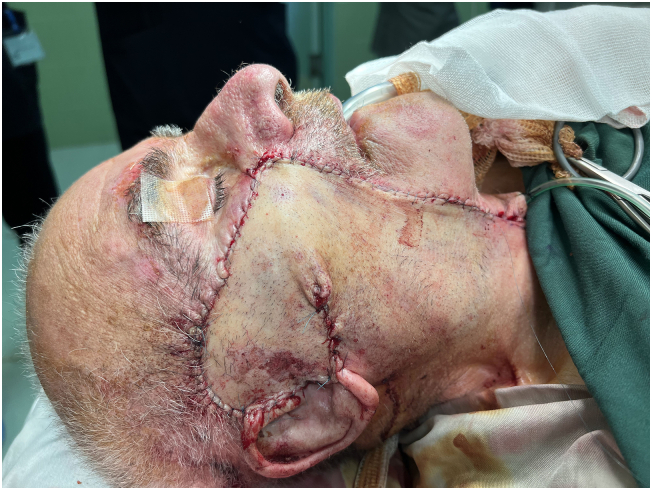
The left-side lesion.

**Fig. 4:**
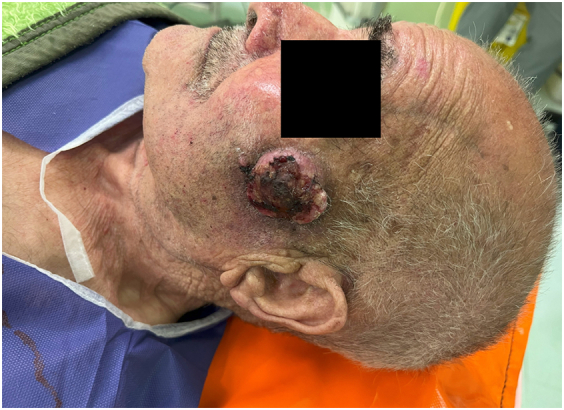
Primary repair of the left lesion by cervical rotational flap.

**Fig. 5:**
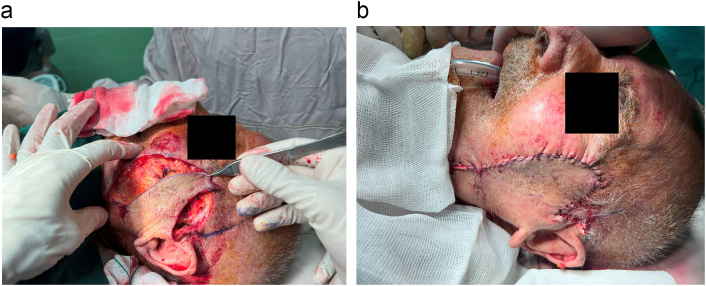
Primary repair of the right lesion by cervical rotational flap.

**Fig. 6:**
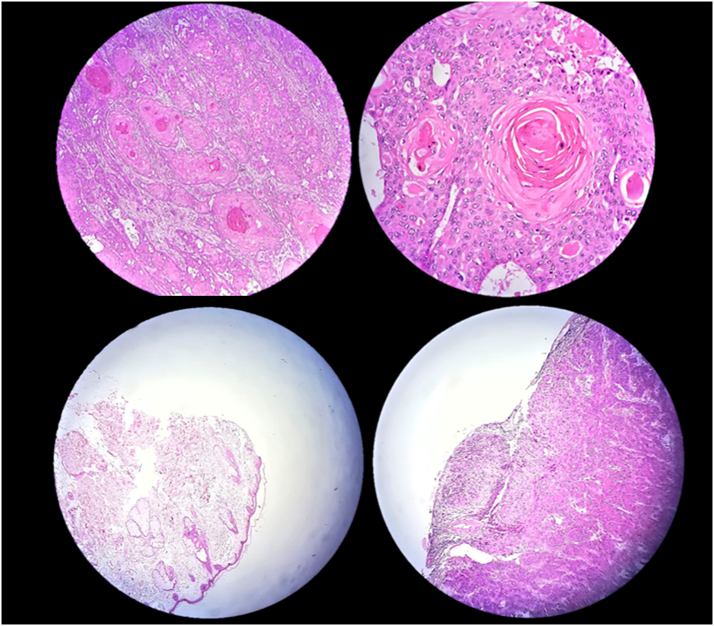
The Haematoxylin and Eosin histopathologic view of the lesions with ×100 in the upper left and ×400 in the upper right reveling “Keratin pearls” competitive with invasive squamous cell carcinoma; superficial margin free of neoplastic cells in the lower left and deep involved margin in the lower right.

An 89-year-old rural male was presented to the plastic and reconstructive surgery ward complaining of the two rather vast and bulging round bizarre wounded masses in the bilateral parotid regions. He had farmed for more than seven decades without sun protection at all. In the patient's past medical history, there was a gait disturbance for three months before and a coronary artery disease ten years ago. In the incisional biopsy taken, invasive squamous cell carcinoma had been reported. After the allowance of the neurologist and cardiologist regarding general anesthesia and operation tolerance, he was scheduled for excision surgery. In the operating room, the right parotid lesion measuring 7.5 × 7.5 cm in dimeters, which was extended to the auricle [Fig. 1], was excised accompanied by superficial parotidectomy [Fig. 2] then using a tensionless Ferguson Cervicofacial skin flap, the cutaneous and subcutaneous layers were reconstructed [Fig. 3]. The health of vascular and nervous structures not involved by the tumour and complete hemostasis was ensured. After the fixation of a removal drain, the latter 5 × 5 cm contralateral lesion [Fig. 4] was removed and reconstructed by a skin flap in a similar method [Fig. 5]. The specimen was sent to the pathology, which was compatible with the biopsy with the details of moderately differentiated SCC; 6 cm and 1 cm tumour size and thickness in their greater diameter, respectively; all surgical margins were free of tumour however, the tumour was located close to deep margin (0.2 cm far); the perineural invasion was present; the vascular invasion was not identified; solar elastosis was present in the right and SCC; moderately differentiated with acantolythic area; tumour size in its greater dimension was 4.2 cm and 1.2 cm greater thickness it had been reached to deep margin; other surgical margins were free of tumour, perineural invasion was also detected; however, the vasculature was intact besides solar elastosis in the left lesion [Fig. 6]. The patient was immediately sent to the intensive care unit. His weekly follow-up for a month and monthly showed no evidence of gross pathologies.

## 3 Discussion

### 3.1 Epidemiology

Regarding the latest Global Cancer Observatory (GLOBOCAN) estimates (2020), HNSCC stands as the seventh most common cancer globally, accounting for an estimated 890,000 new cases (roughly 4.5 % of all cancer diagnoses around the world) and 450,000 deaths per year (approximately 4.6 % of global cancer deaths). Furthermore, its incidence tends to rise and is anticipated to increase by 30 % (that is, 1.08 million new cases annually) by 2030 [[6], [7], [8]]. Generally, the male gender is at 2–4-fold higher risk than the female for developing HNSCC [9]. Non-virally associated HNSCC median age of diagnosis is 66 years, whereas the median age of diagnosis for HPV-associated oropharyngeal cancer and Epstein–Barr virus (EBV)-associated nasopharyngeal cancer is around 53 years and about 50 years, respectively [10,11]. In a cohort of 36 patients with radiographically defined N3 disease with a median follow-up period of 23.6 (range 2.8–135.0) months, the overall survival was 60 % at 2 years and 30 % at 5 years [12]. This project also concluded that no factors were predictive of treatment assignment nor factors associated with overall survival, local and regional control, or distant metastases free-survival on univariate analysis. Compared to mucosal SCC, cutaneous SCC rarely metastasized, varying from 0.5 to 10 %, according to the reports [13,14]. Assuming the reports, the incidence of non-melanoma skin cancer is rising and is estimated to do so until 2040 [15].

### 3.2 Staging

Subdividing primary cutaneous SCC into low and high-risk types has been of interest over the last decade since they behave differently regarding regional metastasis, resulting in prognosis differences [16]. Most cutaneous SCC includes previously untreated small (less than 2 cm), thin (less than 2 mm), and well-differentiated cases, and nodal metastasis is rare [17]. However, a small subset of these tumours, up to 3.7 % to 5.2 %, have a high risk of poor outcomes as developing metastases and a 2.8 % mortality rate [[18], [19], [20], [21]]. 70 % of mortality rates are due to unresectable locoregional disease of note local and nodal involvement rather than distant organ metastases [19]. The American Joint Committee on Cancer (AJCC), in its 7th edition, conducted a tumour classification, introducing several prognostic factors rather than only anatomic features besides tumour diameter [22]. However, AJCC staging could not validate and refine the system via population-based data since CSCC is excluded from the Surveillance, Epidemiology, and End Results Program. The Brigham and Women's Hospital (BWH) alternative tumour classification system is another staging system. An analysis of a single institution cohort has shown that the BWH staging system offers improved distinctiveness, homogeneity, and monotonicity over AJCC 7 [23]. The AJCC Cancer Staging Manual, 8th Edition (AJCC 8) was introduced in January 2018 and included an updated CSCC tumour classification for cases involving only the head and neck [24]. In the study of Roshcer et al. comparing AJCC 8 and BWH tumour classification systems, 103 CSCC tumours with metastasis and 81 without metastasis were included. The authors concluded that neither AJCC8 nor BWH adequately risk-assessed CSCC tumours [25].

### 3.3 Management

Local excision in the large group of low risk can be sufficient and curable [26]. In general, in the UK, SSCC may metastasize in up to 5 % of patients [21]. However, among the high-risk types, the rates have been reported to be much higher, as 33 % in those with poorly differentiated lesions, 45 % in those with lesions more than 4 mm thick, and 47 % in cases of perineural invasion [14]. Elective treatment of the first layer of lymph nodes is a reasonable approach in high-risk patients. However, there is no consensus on managing the N zero nodal basin in CSCC. According to the systematic review and meta-analysis of Thompson et al. paying risk factors for recurrence, metastasis, and disease-specific death in CSCC, nodal metastasis was the single most consistent poor prognostic factor. Invasion beyond the subcutaneous fat (relative risk [RR], 11.21; 95 % confidence interval [CI], 3.59–34.97); Breslow thickness exceeding 6 mm (RR, 6.93; 95 % CI, 4.02–11.94); diameter > 20 mm (RR, 6.15; 95 % CI, 3.56–10.65); poorly differentiated tumours (RR, 4.98; 95 % CI, 3.30–7.49); presence of perineural invasion (RR, 2.95; 95 % CI, 2.31–3.75); immunosuppression (RR, 1.59; 95 % CI, 1.07–2.37); and location on the temple (RR, 2.82; 95 % CI, 1.72–4.63), ear (RR, 2.33; 95 % CI, 1.67–3.23), and lip (RR, 2.28; 95 % CI, 1.54–3.37) were also wholly statistically meaningful for higher risk of nodal metastasis [13].

All of the factors mentioned above, except perineural invasion (PNI) and thickness, are post-operatively assessable. Although Sentinel lymph node (SLN) biopsy is a standard accepted procedure in some malignancies, such as melanoma, its role in CSCC is evolving. From this aspect, the majority of metastasis of head and neck CSCC occurs in the parotid gland as the first echelon of lymph node metastasis, and the neck excision with wide surgical margins accompanied by superficial or total parotidectomy with neck dissection is suggested to be considered in all patients with intra-parotid and neck nodal metastasis. The National Comprehensive Cancer Network (NCCN) has not explicitly recommended a definite guideline for elective treatment of regional nodal basins. Thus, the elective treatment of the parotid and neck is still controversial. However, caution is required to consider superficial parotidectomy with elective neck dissection in high-risk patients. The metastasis rates are highest among patients with poorly differentiated tumours, PNI, thickness>6 mm, immunosuppression, recurrent tumours, and those located in high-risk areas such as the temple and ear [27]. Regarding this, Veness et al. conducted a prospective study involving 266 patients. They concluded that the high risk for metastasis grows when patients present with cSCC of more than 4 to 5 mm in thickness and are located in close proximity to the parotid [28].

Kovatch et al. performed a retrospective chart review study on the outcomes of cutaneous periauricular squamous cell carcinoma (SCC) patients undergoing treatment from 2000 to 2016.

From a total of 112 patients with a median follow-up of 24.5 months, a mean 6 SD age of 75.7 6 10.6 years, there was a strong male predominance (93.8 %). The 26 (23.2 %) patients suffered from preauricular involvement. Patients were primarily treated surgically with primary tumour resection ranging from wide local excision to lateral temporal bone resection (6 parotidectomy and neck dissection), with 17.0 % and 5.4 % receiving adjuvant radiation and chemoradiation, respectively. The metastatic spread was seen to the parotid (25.9 %) and neck (26.8 %), with the most common cervical spread to level II. Nodal disease was associated with worse disease-specific survival (P\.001) and disease-free survival (*P* = .042); pre- and postauricular sites were associated with worse overall survival (*P* = .007) relative to auricular sites [29].

CSCC treatment aims to excise the tumour entirely with minimal functional and cosmetic impairment [30]. Tumour risk and comorbidities determine the treatment. Obtaining this goal requires identifiable tumour margins [31]. Thus, it can be difficult to completely resect such a tumour with indistinct margins in case of poorly defined tumour edges. The recommended safe margins are 4 mm for low-risk tumours of <2 cm in diameter with a well and easily demarcated border and ≥6 mm for high-risk ones [32]. To achieve an acceptable cure rate, Borland et al. suggest that at least a 6-mm excision margin for high-risk SCC is required, compared to the smaller 4–6 mm margin for the low-risk lesions [33]. However, determining whether the resected area is completely clean of cancerous cells without additional reassurance procedures has remained a challenge. The overall prognosis is excellent for most cases: an overall five-year cure rate of >90 % and an overall metastasis rate of 2–5 % [34]. However, tumours with a size over 2 cm double the risk of SCC recurrence and triple the rate of metastasis compared to the smaller ones [31,35]. Higher recurrence rates follow inaccurate margin obtaining [36].

Since the residual tumour skin should never be left under the flap or a skin graft, recurrence may be hidden from direct visual examination and lead to a delay in re-excision [37].

Preventing facial asymmetry and reconstruction after facial tumour surgery is essential [38].

### 3.4 Surgical reconstruction

Since the face is vital to a person's life, it should be reconstructed considering functional and aesthetic aspects. Despite various flap types and techniques, it remained a challenge to meet the multiple demands. It is claimed that after extensive excision of cancerous lesions on the face, or when skin cancer is located on the 3-dimensional structures of the face, reconstruction with a local flap can be impossible, or surgeons are reluctant to reconstruct defects with a skin graft because of postoperative contraction, hyperpigmentation, or other complications. Park et al. reported a case series of eight patients suffering from facial skin cancers underwent complete excision and reconstruction with an arterialized venous-free flap. They evaluated the cosmetic results using ordinary scale methods on the basis of 4 categories of colour, contour, texture, and distortion of surrounding structures besides physical recurrence and metastases of skin cancer. In their average follow-up of 33 months, the whole of the soft-tissue defects made by excising the tumour were reconstructed with good outcomes, except for 1 case, and from the point of the cosmetic evaluation, the colour was fair, the contour and texture were pleasing, absence of distortion of surrounding structures was excellent, and the overall results in most all cases were good. There were no recurrences or metastases during the follow-up period. Finally, they introduced the arterialized venous free flap as an alternative plan among several reconstruction methods when skin cancer on the face is extensively excised [39]. In the study of Lee et al. aiming for the free flap reconstruction of facial defects after SCC excision, including 14 patients from January 2021 to June 2023, all free flaps survived well except one case of partial flap necrosis. Most patients gained good to excellent functional and aesthetic results. The donor site healed uneventfully in all patients. They concluded that free flap reconstruction is an excellent choice for comprehensive skin oncologic defects [40]. Kesting et al.'s study reported post-ablative facial NMSC defects reconstructed using remote-free flaps, including radial forearm, scapular, parascapular, and anterolateral thigh flaps in four patients. They all underwent a split-thickness skin graft (STSG) acquired from the retroauricular region to generate a non-cultured autologous epidermal cell (NCAEC) suspension. Then, four months later, the flap surfaces were de-epithelialized, and the NCAEC suspension was sprayed onto the flap surface to improve the mismatch between facial and flap colour. Debulking was also in the procedure. The aesthetic outcome was examined by photography and clinical examination 3, 6, 9, and 12 months after the first operation, and all flaps survived the 11- to 21-month follow-up. The secondary operation was accompanied by a delay in re-epithelialization in one case. With no STSG donor-site problems occurring, follow-up photographs showed significant improvements in the colour and texture of the flaps. Finally, they concluded that facial reconstruction with free-flap results in a mismatch of colour and texture; however, secondary correction of the flap surface by de-epithelialization and NCAEC application significantly improves the aesthetic outcome [41].

## 4 Conclusion

In our case, some of the macroscopic criteria mentioned were positive: thickness, location on the parotid areas, and right auricular involvement. The surgeon decided to use a palliative surgical approach rather than an utterly curative one. Since the patient was so aged and bedridden and likely could not tolerate much heavier curative and reconstructive operations, the surgeon aimed to excise the tumours as much as he could and reconstruct the fields primarily by a tensionless Ferguson Cervicofacial skin flap.

## Author contribution

Amin Rezazadeh was the surgeon of the patient, planned the surgical technique, and in the role of supervision.

Sonya Sharifi Namin studied the histopathology of the lesions and in the role of reviser.

Ali Samady Khanghah, prepared the manuscript draft as case report and literature review, illustrated the graphics, and in the role of corresponding author to communicate with the journal.

## Consent

Written informed consent was obtained from the patient for publication and any accompanying images. A copy of the written consent is available for review by the Editor-in-Chief of this journal on request.

## Ethical approval

Considering that this is a case report study and does not contain new drug or therapeutic approach trial, the authors assumed that there was no need to take ethical code. The corresponding author on behalf of all authors guarantees the principles of confidentiality. If needed, they can prepare an ethical approval from Research Ethics Committee of Ardabil University of Medical Sciences, Ardabil, Iran.

## Guarantor

Ali Samady Khanghah accepts full responsibility for the work and approves the whole process from designing the study to publish.

## Research registration number

-.

## Funding

There is no funding preparing this manuscript.

## Conflict of interest statement

There are no conflicts of interest.
